# The Podocyte in Diabetic Kidney Disease

**DOI:** 10.1100/tsw.2009.133

**Published:** 2009-10-14

**Authors:** Erin Stitt-Cavanagh, Laura MacLoed, Chris R.J. Kennedy

**Affiliations:** ^1^ Kidney Research Centre, Chronic Disease Program, Ottawa Hospital Research Institute, Canada; ^2^ Department of Cellular and Molecular Medicine, University of Ottawa, Canada

**Keywords:** podocyte, diabetic kidney disease, albuminuria

## Abstract

Approaching epidemic levels, diabetic kidney disease (DKD) is now the leading cause of end-stage renal disease (ESRD). Microalbuminuria is an early clinical marker of DKD that results from damage to the glomerular filtration barrier at the level of the highly differentiated glomerular podocyte cells. Injury to these epithelial cells, podocytopathies, includes cellular hypertrophy, foot process effacement, detachment from the glomerular basement membrane, and apoptosis. Here we review the role of a number of recently identified factors that contribute to podocytopathies in DKD. These factors include members of the renin-angiotensin system (RAS), including angiotensin-converting enzyme (ACE) types 1 and 2, prorenin and its receptor, reactive oxygen species (ROS), prostanoids, peroxisome proliferator-activated receptors (PPAR), advanced glycation end-products (AGEs) and their receptors (RAGE), adiponectin, and microRNAs. As the number of therapeutic options that slow, but do not halt, the progression of DKD to ESRD remains limited, a more comprehensive understanding of the signaling events that contribute to this increasingly prevalent disease may identify novel avenues for treatment and prevention.

### INTRODUCTION: DIABETIC KIDNEY DISEASE

Diabetes mellitus (DM) now accounts for more cases of end-stage renal disease (ESRD) than any other cause of chronic kidney disease (CKD). In North America, four out of every 10 new cases of ESRD arise due to diabetic kidney disease (DKD), a proportion that has risen steadily over the past few decades and shows no signs of slowing. Roughly 30% of individuals with type 1 and 10% of those with type 2 diabetes will develop DKD[1]. Glycemic control along with currently available pharmacotherapies slow, but do not stop, the progression of DKD towards ESRD[2,3,4,5]. For these patients, the only available solution is renal replacement therapy (i.e., transplantation or dialysis). Given the epidemic levels of obesity and type 2 DM in the populations of many Western nations, a comprehensive understanding of the etiology of DKD is urgently needed so that novel therapies can be developed. Along these lines, many efforts are being aimed at identifying the earliest signs of DKD so that treatments can be instituted before irreversible renal injury occurs. Indeed, it is now widely recognized that proteinuria, specifically microalbuminuria, is one of the earliest clinically identifiable markers of diabetes-induced renal damage[6,7,8,9].

### PROTEINURIA IN DIABETIC KIDNEY DISEASE

The appearance of protein in the urine (predominantly the 67-kDa albumin) indicates a compromised glomerular filtration barrier. A strong correlation has been identified between the likelihood of progression to ESRD and the level of albuminuria. Albuminuria is now considered a continuous variable[10] in that even quantities of albumin that are in the upper end of what is considered the "normal" range (<30 mg/day) are associated with an increased risk for the development of later-stage CKD[11,12,13]. Not only does proteinuria predict the pace of renal decline, it is also an indicator of cardiovascular disease (CVD) progression[14]. Indeed, individuals with chronic kidney diseases, such as DKD, presenting with a high urinary protein excretion rate advance more quickly to ESRD than do those with low proteinuria[15]. Proteinuria-lowering interventions, such as angiotensin-converting enzyme inhibitors (ACEi) and angiotensin II receptor blockers (ARBs), along with strict glycemic control, are associated with a slower deterioration of renal function and a delayed onset of ESRD in those with established DKD[16]. However, recent clinical trial data may have tempered enthusiasm for rennin-angiotensin system (RAS) blockade as a means for preventing DKD[17]. Mauer and colleagues showed that early RAS blockade with either losartan or enalapril were unable to prevent albuminuria or slow the progression of nephropathy in normalbuminuric patients with type 1 diabetes despite a significant reduction in the incidence of retinopathy. It is therefore possible that both the choice of target and the timing of the intervention are critical for effective prevention of DKD.

It is thought that proteinuria is not simply an indicator of glomerular damage, nor just a predictor of CVD/CKD progression, but has causative effects on these disease processes[15]. Excessive albumin levels likely overwhelm the metabolic capacity of the renal tubules, thereby promoting inflammation leading to tubulointerstitial fibrosis and an ensuing reduction of glomerular filtration rate (GFR). Intervention prior to these events is critical since tubulointerstitial and glomerular scarring that account for this decline in renal function are currently irreversible. The ensuing activation of the RAS has well-known effects on the vasculature and therefore contributes to the increased CVD risk. Additionally, the appearance of albuminuria may reflect widespread vascular endothelial glycocalyx dysfunction that may manifest similarly in the glomerulus[18]. However, the available ultrastructural evidence showing glomerular endothelial cell injury in the progression of DKD is currently lacking. Taken together, this emerging picture of DKD has made the identification of the cellular loci that yield proteinuria an important goal. Numerous studies over the past decade have increasingly pointed to the podocyte as a key target of injury at the outset of DKD progression (as reviewed in [19,20,21]).

### PODOCYTOPATHIES IN DIABETIC KIDNEY DISEASE

Despite some recent studies that question the role of the glomerular capillary as the primary means for maintaining serum albumin levels[22], most data continue to point towards this remarkable architectural structure as the essential renal filtration sieve. According to this classical view, passage of albumin into Bowman's space is overwhelmingly impeded by the size- and charge-selective glomerular filtration barrier consisting of three interdependent layers: the endothelial cells, the glomerular basement membrane (GBM), and the podocytes. The form and function of the glomerular filtration barrier has been reviewed extensively elsewhere[23,24,25]. The emerging picture is that the morphologically intricate podocyte and its slit diaphragm structure are of primary importance to the maintenance of the filtration barrier, and that injury to this cell is a key event in the initiation of DKD.

Over the past few decades, mesangial cells and the GBM have tended to be the focus of many DKD studies. While glomerular hypertrophy, mesangial matrix expansion, and GBM thickening are classical hallmarks of diabetic glomerular lesions, studies of diabetic patients and animal models reveal that the onset of albuminuria is most closely associated with podocytopathies, such as foot process effacement, podocyte hypertrophy, detachment, apoptosis, and perhaps epithelial-to-mesenchymal transition (EMT)[21]. The well-documented characteristics of diabetes-induced podocyte injury have been comprehensively reviewed elsewhere[19,20,21]. Therefore, we will only briefly highlight some key aspects of podocytopathies, while focusing on the most recent discoveries.

In human and experimental DKD, both foot process effacement and a decreased number of podocytes per glomerulus upon biopsy are well documented[26]. Experimental evidence supporting these clinical observations is abundant. During the early stages of the Akita model of type 1 DKD, as well as the leptin receptor–deficient db/db mouse model of type 2 DKD, podocytes lose nephrin expression, become effaced, and detach from the GBM or undergo apoptosis, events that correlate with the emergence of albuminuria[27]. Similarly, more recent studies of OVE26 transgenic mice, a model of type 1 DKD, show reduced podocyte number and density, although such changes are only evident following the onset of microalbuminuria and may have emerged subsequent to more subtle podocyte injury (e.g., foot process fusion)[28]. Irrespective of these minor differences, podocyte effacement and loss appear to be key events in the early progression of DKD.

Another possible route of podocyte loss in DKD is EMT. During this process, epithelial cells undergo morphological changes, resulting in loss of cell-cell contacts, alteration in cell polarity, and reorganization of the actin cytoskeleton. Cells often revert to an immature undifferentiated phenotype reminiscent of an earlier developmental stage. Transforming growth factor beta (TGF-β), a potent fibrogenic cytokine and EMT inducer, is up-regulated in the glomeruli and nephron segments in a number of kidney diseases, including DKD (as reviewed by [29]). Studies by Li et al. suggest that in podocytes, the actions of TGF-β suppress expression of key slit diaphragm proteins, induce extracellular matrix protein expression (e.g., fibronectin and collagen I), and lead to secretion of matrix metalloproteinase-9 (MMP-9). Together, these changes are consistent with the hypothesis that podocyte EMT contributes to a defective glomerular filtration barrier and albuminuria under pathological conditions[30]. More recently, Yamaguchi et al. assessed a role for EMT in DKD progression[31]. They examined levels of TGF-β–induced fibroblast-specific protein 1 (FSP1), a member of the calmodulin S100 troponin C superfamily, and a fibroblast marker in human DKD[31,32,33]. It was previously shown that tubular epithelial cells overexpressing FSP1 displayed reduced cell adhesion, along with up-regulation of cytokeratin and vimentin[32,34]. Using renal biopsy samples and urine sediment from patients with type 2 DM, they showed that FSP1 levels in podocytes were associated with macroalbuminuria, greater podocyte detachment, and more severe glomerular pathology[31]. Thus, an EMT-like phenotype may arise in podocytes during DKD progression, resulting in detachment from the GBM and promoting podocytopenia.

### MEDIATORS OF PODOCYTE INJURY IN DIABETIC KIDNEY DISEASE

A picture is now emerging of DKD-associated podocytopathies and how they account for alterations to the glomerular filtration barrier. Along with this more comprehensive understanding of the predominant role played by podocyte injury in DKD progression, additional studies aimed at identifying the mechanisms that give rise to these debilitating end-points have likewise contributed invaluable insights. The importance of this field to the nephrology research community is evident from the number of comprehensive reviews provided over the past few years, including, most recently, those discussing the role of vascular endothelial growth factor (VEGF)[35], mechanical stress[36], the NOTCH pathway[37] and TGF-β[38]. For the remainder of this review, we will therefore focus our attention on recent advances of our understanding of several other important mediators of podocyte injury in DKD (summarized in Fig. 1).

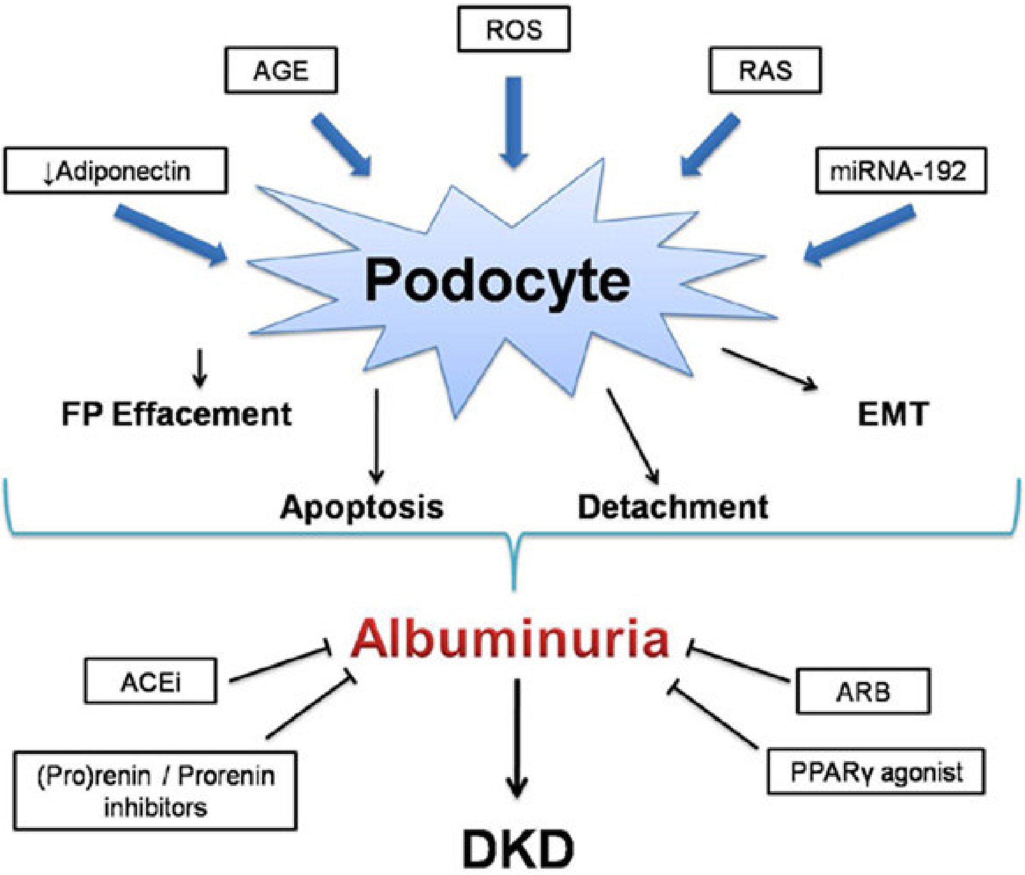

**FIGURE 1.** Mediators of podocyte injury in DKD. Several molecular pathways are known to contribute to podocyte injury in DKD. As depicted, these include, but are not limited to, the following: decreased adiponectin, production of advanced glycation end-products (AGEs), increased reactive oxygen species (ROS), activation of the RAS, and miRNA-192 all contribute to podocyte injury. Podocyte injury exhibits itself as foot process (FP) effacement, apoptosis, detachment, and EMT. Damage to the podocyte leads to increased albuminuria and exacerbates DKD. Agents known to inhibit this process include ARBs, ACEi, peroxisome proliferator-activated receptors (PPAR) agonists, and (pro)renin/prorenin inhibitors.

### The Renin-Angiotensin System

The RAS has been implicated in the progression of DKD. ACEi and ARBs slow DKD progression[3,39,40,41]. For many years, ACEi and ARBs were thought to improve the clinical outcome in diabetic patients via their blood pressure–lowering effects, acting, in part, to mitigate hyperfiltration-enhanced glomerular capillary pressure (Pgc). Indeed, the podocyte is susceptible to the mechanical forces brought about by elevated Pgc. Several studies showed that *in vitro* mechanical stretch, a mimic of elevated Pgc encountered *in vivo*, rendered podocytes more susceptible to a number of injurious end-points, such as apoptosis and actin cytoskeleton reorganization[42,43,44,45], but these studies also led to subsequent discoveries of a podocyte-based, locally acting RAS that appears to induce apoptosis and TGF-β mRNA[42]. Under conditions that reproduce a diabetic milieu (e.g., high glucose, mechanical stretch), podocytes are driven to express several RAS components, including angiotensinogen, as well as both prorenin and AT1 receptors[42,46,47]. Furthermore, angiotensin I and II levels appeared to be enhanced by high glucose. Moreover, recent work by Xu et al., showing that inhibition of the 12-lipoxygenase pathway reduces AT1 expression, lowers proteinuria, and decreases glomerular hypertrophy in a rat streptozotocin (STZ) model of type 1 DM, is consistent with the notion that a local glomerular RAS is directly implicated with filtration barrier injury[48]. Finally, results from recent clinical trials revealed that combined administration of losartan and the rennin-selective inhibitor aliskiren reduce proteinuria in patients with type 2 DM, independent of the blood pressure–lowering effects of the drugs[49].

ACE2 is a novel monocarboxypeptidase member of the RAS family. As a homologue of ACE, it catalyzes the conversion of angiotensin II to Ang-(1-7). It appears that ACE2 activity buffers the deleterious actions of angiotensin II by limiting its levels and via the signaling of Ang-(1-7) through its cell surface Mas receptor[50]. A number of immunohistochemical studies of human and rodent cortical sections have localized ACE2 and the Mas receptor in podocytes and mesangial cells. Interestingly, glomerular ACE2 levels appear to be reduced in human[51,52] and rodent models of DKD[53,54,55], which may allow for enhanced AT1 signaling in podocytes. The impact of Ang-(1-7) signaling and ACE2 activity on the known podocytopathies remains incompletely understood. However, administration of the ACE2 inhibitor MLN-4760 to either db/db mice[55] or STZ-induced diabetic mice[56], as well as targeted deletion of the ACE2 gene[57,58], exacerbates albuminuria. Taken together, this suggests that the glomerular cells, including mesangial cells and podocytes, may be an important renoprotective ACE2 activity locale.

Another potential therapeutic DKD target is the (pro)renin receptor. First identified in 2002, the (pro)renin receptor is highly expressed in the podocyte and can bind both renin and prorenin[59]. An interesting feature of the (pro)renin receptor is that binding of prorenin results in activation of two independent pathways: an angiotensin II–dependent pathway as well as an angiotensin II–independent, (pro)rennin receptor–dependent, intracellular, mitogen-activated protein kinase (MAPK) pathway[60]. Activation of the MAPK pathway results in increased phosphorylation of p42/44 MAPK[61]. To determine the predominant pathway in DKD, Ichihara and coworkers[60] studied both STZ-induced and db/db models of type 1 and type 2 DKD, respectively. The handle region of the (pro)renin receptor that binds the receptor and blocks prorenin activity was administered to diabetic animals. Overexpression of the (pro)renin receptor handle region peptide significantly inhibited the DKD progression and was more effective than ACEi in diabetic angiotensin II type 1a receptor–deficient mice[60]. Similar findings were reported when STZ rats were infused with the handle region peptide[60]. Altogether, these results suggest an important role for prorenin and the (pro)renin receptor in slowing the progression of DKD, and could prove a useful therapeutic target.

### Reactive Oxygen Species

Within the kidney, as in other organs and tissues (i.e., vasculature), hyperglycemia and RAS activation promote oxidative stress, defined as damage to macromolecules caused by ROS (i.e., $O_2^-$, $H_2O_2$, NO, and $ONOO^-$)[62,63]. Nicotinamide adenine dinucleotide phosphate (NADPH) oxidase, or Nox, is a rich source of ROS production in the kidney and indeed within the podocyte[64,65,66,67]. Nox catalyzes production of $O_2^-$ by the one electron reduction of $O_2$ using NADH or NADPH as the electron donor: $2O_2 + NAD(P)H \rightarrow 2{\cdot}O_2^- + NAD(P)^+ + H^+$. Seven Noxes have been identified (Nox1-5, Duox1, and Duox2). Although ROS generation undoubtedly has pleiotropic effects along the nephron and renal vasculature, a role for ROS in damaging the glomerular filtration barrier and, more specifically, the podocyte, is beginning to emerge from the experimental data. Inhibition of NADPH activity by apocynin in mice with STZ-induced diabetes reduces albuminuria, strongly suggesting a role for ROS in glomerular injury associated with type 1 DKD[27]. The identity of specific Nox isoform(s) accounting for glomerular ROS generation awaits confirmation. However, of the Nox isoforms, Nox4 is most abundantly expressed in the kidney and hence was originally termed renal oxidase (Renox)[68]. Nox4, which localizes within the renal vasculature, mesangial cells, tubular cells, and podocytes, appears to be the major renal ROS source and may therefore play a critical role in oxidative stress associated with kidney disease[63]. Recent *in vivo* studies support a role for Nox4 in DKD. In STZ-treated mice, mRNA expression of Nox4 was increased and renal production of ROS was enhanced[69]. A causative relationship between Nox4-induced ROS production and DKD was demonstrated in STZ-induced diabetic rats that were treated with antisense oligonucleotides to reduce Nox4 expression[64]. In these Nox4 antisense-infused animals, renal ROS production and albuminuria were reduced[64]. While these studies provide compelling initial evidence for a role for Nox4 in type 1 DKD, a comprehensive examination of Nox4 expression and downstream effects within podocytes has not been performed.

The notion that podocyte-derived ROS are implicated in DKD was obtained in studies using db/db and Akita mice, where inhibition of Nox activation blunted podocyte apoptosis and detachment, lowered proteinuria, and reduced mesangial matrix expansion[70]. Similar findings were more recently reported in the OVE26 model of type 1 DKD[71]. In these studies, exposure of cultured mouse podocytes to high glucose promoted Nox-induced ROS generation, along with Nox1 and 4 up-regulation. Similarly, Nox1 and 4 mRNA and protein expression were elevated in glomeruli of OVE26 mice. Whether one or both of these Nox isoforms contributes to the deleterious production of ROS in podocytes awaits investigation. Additionally, ROS generation in the podocyte may have other deleterious roles in addition to its proapoptotic actions. ROS could induce actin filament polymerization, leading to cytoskeletal dysfunction and resulting in structural changes to foot processes and slit diaphragms. Furthermore, podocytes exposed to high glucose generate ROS via Nox activity in a cytochrome P450–dependent manner. Treatment of OVE26 diabetic mice with N-hydroxy-N'-(4-butyl-2-methylphenol) formamidine (HET0016), an inhibitor of CYP4A, blocked Nox activity and expression, while reducing albuminuria [71].

In addition to NADPH-dependent generation of ROS, the nitric oxide (NO) pathway has been implicated in the kidney as an important regulator of vascular tone and glomerular ultrafiltration coefficient. NO is generated from L-arginine via the enzyme nitric oxide synthase (NOS). There are three isoforms of NOS: neuronal (nNOS), inducible (iNOS), and endothelial (eNOS). Significantly, eNOS-deficient db/db mice display severe glomerular lesions and albuminuria[72]. NO may abrogate VEGF-mediated macrophage migration in DKD via inhibition of Flt-1 VEGF receptor expression, and its loss via eNOS deletion may contribute to glomerular injury[73]. However, further studies are required to determine whether podocytes are involved in the progression of this glomerular injury subsequent to local NO production.

### Cyclooxygenases and Prostanoids

The prostanoids are comprised of prostaglandins E2, F2α, I2, and D2, along with thromboxane A2. These lipid mediators are derived from arachidonic acid via the sequential catalytic activities of cyclooxygenases (COX), of which there are two isoforms, COX-1 and COX-2, followed by that of specific synthases PGE, PGF, PGI, PGD, and thromboxane synthase. We have known for decades that COX inhibitors (nonsteroidal anti-inflammatory drugs, NSAIDs), which block prostanoid synthesis, reduce proteinuria in a number of glomerulopathies[74,75,76,77,78,79,80], including DKD[81]. Although COX inhibition delivers antiproteinuric effects, NSAIDs, including the gastrointestinal-sparing COX-2 selective inhibitors, can be nephrotoxic for CKD patients[82,83]. By blocking the synthesis of vasodilatory prostaglandins, these drugs can elicit a precipitous decline in renal blood flow and GFR[84,85,86,87,88]. Clearly, if the beneficial proteinuria-lowering effects of COX inhibition are to be realized, we will need to identify the cellular targets that modulate the antiproteinuric effects of COX inhibitors. Furthermore, strategies must move downstream of COX blockade and differentiate between those prostanoids and their respective receptors, which deliver protective effects from those that impair renal function.

COX-2 inhibitors decrease proteinuria and reduce the severity of glomerular lesions in STZ-induced DKD[89]. In several experimental models, as well as in human biopsy specimens, COX-2 expression becomes detectable in podocytes during early disease[90,91,92,93]. These data suggest that in addition to their effects on the renal vasculature, prostanoids may have direct interactions with the cells of the glomerular filtration barrier. Indeed, prostanoids act locally from their sites of synthesis to elicit a variety of actions via a set of classical G-protein coupled receptors (GPCRs). PGE2 and TXA2 are major renal prostanoids whose actions are mediated via specific GPCRs known as the E and T-Prostanoid receptors

(EP1-EP4, TP), respectively. The mesangium[94,95] and podocytes[96] express a number of prostanoid receptors, including EP1, EP4, and TP receptors, among others. EP1 receptors may modulate maladaptive aspects of DKD, such as glomerular hypertrophy, matrix expansion, TGF-β production, and proteinuria[97]. Makino and colleagues showed that an EP1 antagonist diminished glomerular damage and proteinuria in rats with STZ-induced type 1 diabetes[97]. Interestingly, glomerular expression of both EP1 and EP4 receptor mRNA both increased in these STZ rats. We have likewise observed enhanced glomerular EP4 and TP receptor mRNA levels in db/db mice (unpublished results). In addition, we recently uncovered a novel feedback loop in podocytes whereby an *in vitro* surrogate for glomerular capillary pressure (i.e., equibiaxial mechanical stretch), along with PGE2 stimulation of the prostaglandin EP4 receptor, induces COX-2 in a p38 MAPK-dependent manner, resulting in actin cytoskeletal rearrangements[45,98]. We speculate that PGE2-dependent stimulation of the EP4 receptor in podocytes exacerbates glomerulopathies, such as DKD, that are associated with enhanced Pgc.

A role for the TP receptor in modulating the glomerular filtration barrier is suggested by several studies. Thromboxane levels rise in experimental DKD[99] and administration of a TP antagonist markedly reduces albuminuria in rodent models of DKD[100,101,102]. Inasmuch as these studies are consistent with a role for EP and TP receptors in filtration barrier dysregulation, whether podocytes are cellular targets for these deleterious actions of PGE2/TXA2 remains incompletely defined. Furthermore, the precise mechanisms that might account for these effects on the filtration barrier await identification.

### Peroxisome Proliferator-Activated Receptors

A role for PPAR signaling in DKD progression has emerged from a number of studies. Belonging to the nuclear receptor superfamily, PPARs were first discovered by Issemann and Green in 1990[103]. Acting as transcription factors, PPARs modulate gene transcription by heterodimerizing with the retinoic acid receptor α (RXRα). Three PPAR subtypes (α, β, γ) have been cloned and characterized, all of which are encoded by specific genes. Potent synthetic ligands of PPARs are effective therapeutics for dyslipidemia as well as diabetes (reviewed in [104]). Specifically, PPARγ agonism with rosiglitazone improves insulin resistance, hyperinsulinemia, and hyperglycemia[105,106].

Rosiglitazone has a direct and protective effect on glucose uptake in cultured human podocytes via up-regulation of the GLUT1 glucose transporter. This phenomenon was dependent on nephrin expression, as podocytes lacking nephrin were incapable of modulating glucose uptake[107]. Interestingly, studies by Benigni et al. showed that nephrin expression is regulated by the PPARγ agonist pioglitazone, which in a model of Heyman nephritis results in decreased proteinuria[108].

### Advanced Glycation End-Products

AGEs are generated in diabetes through the interaction of glucose with protein or lipid molecules. These molecules have a number of toxic effects throughout the body, including the vasculature and within renal glomeruli, where they accumulate in the mesangium, endothelial cells, GBM, and podocytes of individuals with DKD[109]. The podocyte appears to be a sensitive target of AGE actions as it expresses the receptor for AGE (RAGE)[110]. Accordingly, Doublier et al. demonstrated that glycated albumin inhibited nephrin expression in cultured podocytes through engagement of RAGE, which is consistent with reduced levels of this key slit diaphragm component observed in human and experimental DKD[111]. Blockade of the AGE pathway with anti-AGE agents ALT-711, KIOM-79, and LR-90 slow the progression of DKD in Zucker diabetic fatty rats and db/db mice[112,113,114]. The mechanism of action for anti-AGE agents appears to involve, at least in part, a reduction in glomerular cytokine levels, as KIOM-79 administration to Zucker diabetic fatty rats reduced TGF-β levels and decreased podocyte apoptosis[114]. Other actions of AGEs in the podocyte were identified by Ruster et al., who showed that podocyte exposure to AGE-bovine serum albumin (AGE-BSA) induced activation of the cell cycle regulatory protein p27(Kip1), leading to podocyte apoptosis[115]. Taken together, the AGE/RAGE system appears to contribute significantly to podocyte injury in DKD and could emerge as a definitive therapeutic target.

### Adiponectin

Produced in adipose tissue, adiponectin is an important peptide hormone involved with glucose regulation and fatty acid catabolism. Decreased adiponectin levels result in oxidative stress, fusion of podocyte foot processes, and microalbuminuria[116,117,118]. Consistent with these findings, adiponectin knockout mice have increased susceptibility to podocyte injury in a subtotal renal ablation model of progressive CKD[119]. Based on such studies, some have suggested that adiponectin may be a biomarker for kidney disease and, due to its involvement in protecting the filtration barrier, a useful therapeutic target in slowing DKD progression[120]. Evidence to support a role for adiponectin in podocyte function is accumulating. A recent study by Cammisotto and Bendayan revealed that stimulation of the adiponectin receptor in podocytes yielded activation of AMP-activated protein kinase, which controls oxidative stress and apoptosis[121].

### MicroRNA

MicroRNAs (miRNAs) are single-stranded, noncoding, RNA molecules that regulate gene expression by interfering with protein translation of mRNA targets, resulting in the degradation of mRNA species. The role of miRNAs in the kidney was recently reviewed[122] and, thus, we will only provide a brief overview of this emerging field. Processing of miRNAs is facilitated by the enzyme Dicer, which when deleted from podocytes, leads to filtration barrier dysfunction in mice[123,124,125]. Furthermore, five kidney-specific miRNAs have been identified (miRNA-192, -194, -204, -215, and -216) and are further segregated into two classes: those found predominantly in the cortex and those present in the medulla[126,127]. When investigating the role of miRNA from isolated diabetic glomeruli, Kato et al. showed that miRNA-192 levels were significantly higher in STZ-induced diabetic and db/db mice compared to nondiabetic controls[128]. Elevated miRNA-192 positively correlated with increased TGF-β and collagen 1α2 levels, which may have an impact on GBM thickness and podocyte health[128]. Clearly, our understanding of the role played by various miRNA species in not only the podocytes, but in the kidney as a whole, is at a nascent stage. Undoubtedly, future studies will continue to unravel the complex miRNA-based gene regulation in the context of the podocyte during DKD progression, and it is foreseeable that novel targets will be identified for therapeutic intervention with specific miRNA inhibitors.

### CONCLUSION

The large number of factors that contribute to the etiology of podocyte dysfunction in DKD is indicative of the complexity of this condition. Nevertheless, as the number of therapeutic options that slow, but do not halt, the progression of DKD to ESRD remains limited, a more comprehensive understanding of the underlying signaling events that contribute to this increasingly prevalent disease is needed in order to identify novel avenues for treatment and prevention.

### ACKNOWLEDGMENTS

Erin Stitt-Cavanagh is an Ontario Graduate Scholarship in Sciences and Technology Scholarship awardee.

## References

1. (2005) Incidence of end-stage renal disease among persons with diabetes--United States, 1990-2002. *MMWR Morb. Mortal. Wkly. Rep.* **54(43),** 1097–1100.
2. Wolf, G. and Ritz, E. (2005) Combination therapy with ACE inhibitors and angiotensin II receptor blockers to halt progression of chronic renal disease: pathophysiology and indications. *Kidney Int.* **67,** 799–812.
3. Lewis, E.J. and Lewis, J.B. (2003) Treatment of diabetic nephropathy with angiotensin II receptor antagonist. *Clin. Exp. Nephrol*. **7,** 1–8.
4. Remuzzi, G., Ruggenenti, P., Perna, A., Dimitrov, B.D., de Zeeuw, D., Hille, D.A., Shahinfar, S., Carides, G.W., and Brenner, B.M. (2004) Continuum of renoprotection with losartan at all stages of type 2 diabetic nephropathy: a post hoc analysis of the RENAAL trial results. *J. Am. Soc. Nephrol.* **15,** 3117–3125.
5. Ruggenenti, P., Fassi, A., Ilieva, A.P., Bruno, S., Iliev, I.P., Brusegan, V., Rubis, N., Gherardi, G., Arnoldi, F., Ganeva, M., Ene-Iordache, B., Gaspari, F., Perna, A., Bossi, A., Trevisan, R., Dodesini, A.R., and Remuzzi, G. (2004) Preventing microalbuminuria in type 2 diabetes. *N. Engl. J. Med.* **351,** 1941–1951.
6. Dronavalli, S., Duka, I., and Bakris, G.L. (2008) The pathogenesis of diabetic nephropathy. *Nat. Clin. Pract. Endocrinol. Metab.* **4,** 444–452.
7. Kanwar, Y.S., Wada, J., Sun, L., Xie, P., Wallner, E.I., Chen, S., Chugh, S., and Danesh, F.R. (2008) Diabetic nephropathy: mechanisms of renal disease progression. *Exp. Biol. Med. (Maywood)* **233,** 4–11.
8. Wolf, G. and Ziyadeh, F.N. (1999) Molecular mechanisms of diabetic renal hypertrophy. *Kidney Int.* **56,** 393–405.
9. Wolf, G. and Ziyadeh, F.N. (2007) Cellular and molecular mechanisms of proteinuria in diabetic nephropathy. *Nephron. Physiol.* **106,** 26–31.
10. Ruggenenti, P. and Remuzzi, G. (2006) Time to abandon microalbuminuria? *Kidney Int.* **70,** 1214–1222.
11. Anavekar, N.S., Gans, D.J., Berl, T., Rohde, R.D., Cooper, W., Bhaumik, A., Hunsicker, L.G., Rouleau, J.L., Lewis, J.B., Rosendorff, C., Porush, J.G., Drury, P.L., Esmatjes, E., Raz, I., Vanhille, P., Locatelli, F., Goldhaber, S., Lewis, E.J., and Pfeffer, M.A. (2004) Predictors of cardiovascular events in patients with type 2 diabetic nephropathy and hypertension: a case for albuminuria. *Kidney Int. Suppl.* **(92)**, S50–55.
12. Karalliedde, J. and Viberti, G. (2005) Hypertension and microalbuminuria: risk factors for cardiovascular disease in diabetes. *Curr. Hypertens. Rep*. **7,** 1–2.
13. Karalliedde, J. and Viberti, G. (2004) Microalbuminuria and cardiovascular risk. *Am. J. Hypertens.* **17,** 986–993.
14. Basi, S. and Lewis, J.B. (2006) Microalbuminuria as a target to improve cardiovascular and renal outcomes. *Am. J. Kidney Dis.* **47,** 927–946.
15. Remuzzi, G., Benigni, A., and Remuzzi, A. (2006) Mechanisms of progression and regression of renal lesions of chronic nephropathies and diabetes. *J. Clin. Invest*. **116,** 288–296.
16. Remuzzi, A., Gagliardini, E., Sangalli, F., Bonomelli, M., Piccinelli, M., Benigni, A., and Remuzzi, G. (2006) ACE inhibition reduces glomerulosclerosis and regenerates glomerular tissue in a model of progressive renal disease. *Kidney Int*. **69,** 1124–1130.
17. Mauer, M., Zinman, B., Gardiner, R., Suissa, S., Sinaiko, A., Strand, T., Drummond, K., Donnelly, S., Goodyer, P., Gubler, M.C., and Klein, R. (2009) Renal and retinal effects of enalapril and losartan in type 1 diabetes. *N. Engl. J. Med*. **361,** 40–51.
18. Satchell, S.C. and Tooke, J.E. (2008) What is the mechanism of microalbuminuria in diabetes: a role for the glomerular endothelium? *Diabetologia* **51,** 714–725.
19. Reddy, G.R., Kotlyarevska, K., Ransom, R.F., and Menon, R.K. (2008) The podocyte and diabetes mellitus: is the podocyte the key to the origins of diabetic nephropathy? *Curr. Opin. Nephrol. Hypertens.* **17,** 32–36.
20. Wolf, G., Chen, S., and Ziyadeh, F.N. (2005) From the periphery of the glomerular capillary wall toward the center of disease: podocyte injury comes of age in diabetic nephropathy. *Diabetes* **54,** 1626–1634.
21. Jefferson, J.A., Shankland, S.J., and Pichler, R.H. (2008) Proteinuria in diabetic kidney disease: a mechanistic viewpoint. *Kidney Int.* **74,** 22–36.
22. Russo, L.M., Sandoval, R.M., Campos, S.B., Molitoris, B.A., Comper, W.D., and Brown, D. (2009) Impaired tubular uptake explains albuminuria in early diabetic nephropathy. *J. Am. Soc. Nephrol.* **20,** 489–494.
23. Haraldsson, B., Nystrom, J., and Deen, W.M. (2008) Properties of the glomerular barrier and mechanisms of proteinuria. *Physiol. Rev*. **88,** 451–487.
24. Deen, W.M. (2004) What determines glomerular capillary permeability? *J. Clin. Invest.* **114,** 1412–1414.
25. Deen, W.M., Lazzara, M.J., and Myers, B.D. (2001) Structural determinants of glomerular permeability. *Am. J. Physiol. Renal Physiol.* **281,** F579–596.
26. Steffes, M.W., Schmidt, D., McCrery, R., and Basgen, J.M. (2001) Glomerular cell number in normal subjects and in type 1 diabetic patients. *Kidney Int.* **59,** 2104–2113.
27. Susztak, K., Raff, A.C., Schiffer, M., and Bottinger, E.P. (2006) Glucose-induced reactive oxygen species cause apoptosis of podocytes and podocyte depletion at the onset of diabetic nephropathy. *Diabetes* **55,** 225–233.
28. Teiken, J.M., Audettey, J.L., Laturnus, D.I., Zheng, S., Epstein, P.N., and Carlson, E.C. (2008) Podocyte loss in aging OVE26 diabetic mice. *Anat. Rec. (Hoboken)* **291,** 114–121.
29. Liu, Y. (2006) Renal fibrosis: new insights into the pathogenesis and therapeutics. *Kidney Int.* **69,** 213–217.

30. Li, Y., Kang, Y.S., Dai, C., Kiss, L.P., Wen, X., and Liu, Y. (2008) Epithelial-to-mesenchymal transition is a potential pathway leading to podocyte dysfunction and proteinuria. *Am. J. Pathol.* **172,** 299–308.
31. Yamaguchi, Y., Iwano, M., Suzuki, D., Nakatani, K., Kimura, K., Harada, K., Kubo, A., Akai, Y., Toyoda, M., Kanauchi, M., Neilson, E.G., and Saito, Y. (2009) Epithelial-mesenchymal transition as a potential explanation for podocyte depletion in diabetic nephropathy. *Am. J. Kidney Dis.* **54(4),** 653–664.
32. Strutz, F., Okada, H., Lo, C.W., Danoff, T., Carone, R.L., Tomaszewski, J.E., and Neilson, E.G. (1995) Identification and characterization of a fibroblast marker: FSP1. *J. Cell Biol.* **130,** 393–405.
33. Iwano, M., Plieth, D., Danoff, T.M., Xue, C., Okada, H., and Neilson, E.G. (2002) Evidence that fibroblasts derive from epithelium during tissue fibrosis. *J. Clin. Invest.* **110,** 341–350.
34. Okada, H., Danoff, T.M., Kalluri, R., and Neilson, E.G. (1997) Early role of Fsp1 in epithelial-mesenchymal transformation. *Am. J. Physiol*. **273,** F563–574.
35. Chen, S. and Ziyadeh, F.N. (2008) Vascular endothelial growth factor and diabetic nephropathy. *Curr. Diab. Rep.* **8,** 470–476.
36. Lewko, B. and Stepinski, J. (2009) Hyperglycemia and mechanical stress: targeting the renal podocyte. *J. Cell Physiol*. **221(2),** 288–295.
37. Mertens, P.R., Raffetseder, U., and Rauen, T. (2008) Notch receptors: a new target in glomerular diseases. *Nephrol. Dial. Transplant*. **23,** 2743–2745.
38. Ziyadeh, F.N. (2008) Different roles for TGF-beta and VEGF in the pathogenesis of the cardinal features of diabetic nephropathy. *Diabetes Res. Clin. Pract.* **82(Suppl 1),** S38–41.
39. Philips, J.C., Weekers, L., and Scheen, A.J. (2001) [Clinical study of the month. The CALM study assessing the combination of an angiotensin-converting enzyme inhibitor and an angiotensin II receptor antagonist in the treatment of diabetic nephropathy]. *Rev. Med. Liege* **56,** 126–128.
40. Mifsud, S.A., Allen, T.J., Bertram, J.F., Hulthen, U.L., Kelly, D.J., Cooper, M.E., Wilkinson-Berka, J.L., and Gilbert, R.E. (2001) Podocyte foot process broadening in experimental diabetic nephropathy: amelioration with renin-angiotensin blockade. *Diabetologia* **44,** 878–882.
41. Langham, R.G., Kelly, D.J., Cox, A.J., Thomson, N.M., Holthofer, H., Zaoui, P., Pinel, N., Cordonnier, D.J. and Gilbert, R.E. (2002) Proteinuria and the expression of the podocyte slit diaphragm protein, nephrin, in diabetic nephropathy: effects of angiotensin converting enzyme inhibition. *Diabetologia* **45,** 1572–1576.
42. Durvasula, R.V., Petermann, A.T., Hiromura, K., Blonski, M., Pippin, J., Mundel, P., Pichler, R., Griffin, S., Couser, W.G., and Shankland, S.J. (2004) Activation of a local tissue angiotensin system in podocytes by mechanical strain. *Kidney Int.* **65,** 30–39.
43. Petermann, A.T., Pippin, J., Durvasula, R., Pichler, R., Hiromura, K., Monkawa, T., Couser, W.G., and Shankland, S.J. (2005) Mechanical stretch induces podocyte hypertrophy in vitro. *Kidney Int.* **67,** 157–166.
44. Endlich, N., Kress, K.R., Reiser, J., Uttenweiler, D., Kriz, W., Mundel, P., and Endlich, K. (2001) Podocytes respond to mechanical stress in vitro. *J. Am. Soc. Nephrol.* **12,** 413–422.
45. Martineau, L.C., McVeigh, L.I., Jasmin, B.J., and Kennedy, C.R. (2004) p38 MAP kinase mediates mechanically induced COX-2 and PG EP4 receptor expression in podocytes: implications for the actin cytoskeleton. *Am. J. Physiol. Renal Physiol.* **286,** F693–701.
46. Durvasula, R.V. and Shankland, S.J. (2008) Activation of a local renin angiotensin system in podocytes by glucose. *Am. J. Physiol. Renal Physiol.* **294,** F830–839.
47. Yoo, T.H., Li, J.J., Kim, J.J., Jung, D.S., Kwak, S.J., Ryu, D.R., Choi, H.Y., Kim, J.S., Kim, H.J., Han, S.H., Lee, J.E., Han, D.S., and Kang, S.W. (2007) Activation of the renin-angiotensin system within podocytes in diabetes. *Kidney Int.* **71,** 1019–1027.
48. Xu, Z.G., Miao, L.N., Cui, Y.C., Jia, Y., Yuan, H., and Wu, M. (2009) Angiotensin II type 1 receptor expression is increased via 12-lipoxygenase in high glucose-stimulated glomerular cells and type 2 diabetic glomeruli. *Nephrol. Dial. Transplant.* **24,** 1744–1752.
49. Parving, H.H., Persson, F., Lewis, J.B., Lewis, E.J., and Hollenberg, N.K. (2008) Aliskiren combined with losartan in type 2 diabetes and nephropathy. *N. Engl. J. Med.* **358,** 2433–2446.
50. Dilauro, M. and Burns, K.D. (2009) Angiotensin-(1-7) and its effects in the kidney. *TheScientificWorldJOURNAL* **9,** 522–535.
51. Reich, H.N., Oudit, G.Y., Penninger, J.M., Scholey, J.W., and Herzenberg, A.M. (2008) Decreased glomerular and tubular expression of ACE2 in patients with type 2 diabetes and kidney disease. *Kidney Int.* **74,** 1610–1616.
52. Mizuiri, S., Hemmi, H., Arita, M., Ohashi, Y., Tanaka, Y., Miyagi, M., Sakai, K., Ishikawa, Y., Shibuya, K., Hase, H., and Aikawa, A. (2008) Expression of ACE and ACE2 in individuals with diabetic kidney disease and healthy controls. *Am. J. Kidney Dis.* **51,** 613–623.
53. Tikellis, C., Johnston, C.I., Forbes, J.M., Burns, W.C., Burrell, L.M., Risvanis, J., and Cooper, M.E. (2003) Characterization of renal angiotensin-converting enzyme 2 in diabetic nephropathy. *Hypertension* **41,** 392–397.
54. Leehey, D.J., Singh, A.K., Bast, J.P., Sethupathi, P., and Singh, R. (2008) Glomerular renin angiotensin system in streptozotocin diabetic and Zucker diabetic fatty rats. *Transl. Res.* **151,** 208–216.
55. Ye, M., Wysocki, J., William, J., Soler, M.J., Cokic, I., and Batlle, D. (2006) Glomerular localization and expression of angiotensin-converting enzyme 2 and angiotensin-converting enzyme: implications for albuminuria in diabetes. *J. Am. Soc. Nephrol.* **17,** 3067–3075.

56. Soler, M.J., Wysocki, J., Ye, M., Lloveras, J., Kanwar, Y., and Batlle, D. (2007) ACE2 inhibition worsens glomerular injury in association with increased ACE expression in streptozotocin-induced diabetic mice. *Kidney Int.* **72,** 614–623.
57. Tikellis, C., Bialkowski, K., Pete, J., Sheehy, K., Su, Q., Johnston, C., Cooper, M.E., and Thomas, M.C. (2008) ACE2 deficiency modifies renoprotection afforded by ACE inhibition in experimental diabetes. *Diabetes* **57,** 1018–1025.
58. Wong, D.W., Oudit, G.Y., Reich, H., Kassiri, Z., Zhou, J., Liu, Q.C., Backx, P.H., Penninger, J.M., Herzenberg, A.M., and Scholey, J.W. (2007) Loss of angiotensin-converting enzyme-2 (Ace2) accelerates diabetic kidney injury. *Am. J. Pathol.* **171,** 438–451.
59. Nguyen, G., Delarue, F., Burckle, C., Bouzhir, L., Giller, T., and Sraer, J.D. (2002) Pivotal role of the renin/prorenin receptor in angiotensin II production and cellular responses to renin. *J. Clin. Invest.* **109,** 1417–1427.
60. Ichihara, A., Sakoda, M., Mito-Kurauchi, A., and Itoh, H. (2008) Activated prorenin as a therapeutic target for diabetic nephropathy. *Diabetes Res. Clin. Pract.* **82(Suppl 1),** S63–66.
61. Nguyen, G. (2006) Renin/prorenin receptors. *Kidney Int.* **69,** 1503–1506.
62. Li, J.M. and Shah, A.M. (2003) ROS generation by nonphagocytic NADPH oxidase: potential relevance in diabetic nephropathy. *J. Am. Soc. Nephrol.* **14,** S221–226.
63. Gill, P.S. and Wilcox, C.S. (2006) NADPH oxidases in the kidney. *Antioxid. Redox Signal.* **8,** 1597–1607.
64. Gorin, Y., Block, K., Hernandez, J., Bhandari, B., Wagner, B., Barnes, J.L., and Abboud, H.E. (2005) Nox4 NAD(P)H oxidase mediates hypertrophy and fibronectin expression in the diabetic kidney. *J. Biol. Chem.* **280,** 39616–39626.
65. Kim, N.H., Rincon-Choles, H., Bhandari, B., Choudhury, G.G., Abboud, H.E., and Gorin, Y. (2006) Redox dependence of glomerular epithelial cell hypertrophy in response to glucose. *Am. J. Physiol. Renal Physiol.* **290,** F741–751.
66. Satoh, M., Fujimoto, S., Haruna, Y., Arakawa, S., Horike, H., Komai, N., Sasaki, T., Tsujioka, K., Makino, H., and Kashihara, N. (2005) NAD(P)H oxidase and uncoupled nitric oxide synthase are major sources of glomerular superoxide in rats with experimental diabetic nephropathy. *Am. J. Physiol. Renal Physiol.* **288,** F1144–1152.
67. Greiber, S., Munzel, T., Kastner, S., Muller, B., Schollmeyer, P., and Pavenstadt, H. (1998) NAD(P)H oxidase activity in cultured human podocytes: effects of adenosine triphosphate. *Kidney Int.* **53,** 654–663.
68. Geiszt, M., Kopp, J.B., Varnai, P., and Leto, T.L. (2000) Identification of renox, an NAD(P)H oxidase in kidney. *Proc. Natl. Acad. Sci. U. S. A.* **97,** 8010–8014.
69. Etoh, T., Inoguchi, T., Kakimoto, M., Sonoda, N., Kobayashi, K., Kuroda, J., Sumimoto, H., and Nawata, H. (2003) Increased expression of NAD(P)H oxidase subunits, NOX4 and p22phox, in the kidney of streptozotocin-induced diabetic rats and its reversibity by interventive insulin treatment. *Diabetologia* **46,** 1428–1437.
70. Susztak, K., Raff, A.C., Schiffer, M., and Bottinger, E.P. (2006) Glucose-induced reactive oxygen species cause apoptosis of podocytes and podocyte depletion at the onset of diabetic nephropathy. *Diabetes* **55,** 225–233.
71. Eid, A.A., Gorin, Y., Fagg, B.M., Maalouf, R., Barnes, J.L., Block, K., and Abboud, H.E. (2009) Mechanisms of podocyte injury in diabetes: role of cytochrome P450 and NADPH oxidases. *Diabetes* **58,** 1201–1211.
72. Zhao, H.J., Wang, S., Cheng, H., Zhang, M.Z., Takahashi, T., Fogo, A.B., Breyer, M.D., and Harris, R.C. (2006) Endothelial nitric oxide synthase deficiency produces accelerated nephropathy in diabetic mice. *J. Am. Soc. Nephrol.* **17**, 2664–2669.
73. Sato, W., Kosugi, T., Zhang, L., Roncal, C.A., Heinig, M., Campbell-Thompson, M., Yuzawa, Y., Atkinson, M.A., Grant, M.B., Croker, B.P., and Nakagawa, T. (2008) The pivotal role of VEGF on glomerular macrophage infiltration in advanced diabetic nephropathy. *Lab. Invest.* **88,** 949–961.
74. Golbetz, H., Black, V., Shemesh, O., and Myers, B.D. (1989) Mechanism of the antiproteinuric effect of indomethacin in nephrotic humans. *Am. J. Physiol.* **256**, F44–51.
75. Velosa, J.A. and Torres, V.E. (1986) Benefits and risks of nonsteroidal antiinflammatory drugs in steroid-resistant nephrotic syndrome. *Am. J. Kidney Dis.* **8,** 345–350.
76. Velosa, J.A., Torres, V.E., Donadio, J.V., Jr., Wagoner, R.D., Holley, K.E., and Offord, K.P. (1985) Treatment of severe nephrotic syndrome with meclofenamate: an uncontrolled pilot study. *Mayo Clin. Proc.* **60,** 586–592.
77. Vriesendorp, R., Brentjens, J.R., and Donker, A.J. (1985) Nonsteroidal antiinflammatory drugs in the nephrotic syndrome. *Minerva Urol. Nefrol.* **37,** 211–219.
78. Vriesendorp, R., de Zeeuw, D., de Jong, P.E., Donker, A.J., Pratt, J.J., and van der Hem, G.K. (1986) Reduction of urinary protein and prostaglandin E2 excretion in the nephrotic syndrome by non-steroidal anti-inflammatory drugs. *Clin. Nephrol.* **25,** 105–110.
79. Vriesendorp, R., Donker, A.J., de Zeeuw, D., de Jong, P.E., and van der Hem, G.K. (1985) Antiproteinuric effect of naproxen and indomethacin. A double-blind crossover study. *Am. J. Nephrol.* **5,** 236–242.
80. Vriesendorp, R., Donker, A.J., de Zeeuw, D., de Jong, P.E., van der Hem, G.K., and Brentjens, J.R. (1986) Effects of nonsteroidal anti-inflammatory drugs on proteinuria. *Am. J. Med.* **81,** 84–94.
81. Dunn, M.J. (1990) The roles of angiotensin II and prostaglandins in the regulation of the glomerular filtration of albumin. *J. Hypertens. Suppl.* **8,** S47–51; discussion S51–42.
82. Noroian, G. and Clive, D. (2002) Cyclo-oxygenase-2 inhibitors and the kidney: a case for caution. *Drug Saf.* **25,** 165–172.
83. Perazella, M.A. (2002) COX-2 selective inhibitors: analysis of the renal effects. *Expert Opin. Drug Saf.* **1,** 53–64.

84. Whelton, A. (1999) Nephrotoxicity of nonsteroidal anti-inflammatory drugs: physiologic foundations and clinical implications. *Am. J. Med.* **106,** 13S–24S.
85. Breyer, M.D. and Breyer, R.M. (2001) G protein-coupled prostanoid receptors and the kidney. *Annu. Rev. Physiol.* **63,** 579–605.
86. Epstein, M. (2002) Non-steroidal anti-inflammatory drugs and the continuum of renal dysfunction. *J. Hypertens.* **20(Suppl 6),** S17–23.
87. Blume, C., Heise, G., Muhlfeld, A., Bach, D., Schror, K., Gerhardz, C.D., Grabensee, B., and Heering, P. (1999) Effect of flosulide, a selective cyclooxygenase 2 inhibitor, on passive heymann nephritis in the rat. *Kidney Int.* **56,** 1770–1778.
88. Murray, M.D. and Brater, D.C. (1997) Effects of NSAIDs on the kidney. *Prog. Drug Res.* **49,** 155–171.
89. Cheng, H.F. and Harris, R.C. (2002) Cyclooxygenase-2 expression in cultured cortical thick ascending limb of Henle increases in response to decreased extracellular ionic content by both transcriptional and post-transcriptional mechanisms. Role of p38-mediated pathways. *J. Biol. Chem.* **277,** 45638–45643.
90. Hirose, S., Yamamoto, T., Feng, L., Yaoita, E., Kawasaki, K., Goto, S., Fujinaka, H., Wilson, C.B., Arakawa, M., and Kihara, I. (1998) Expression and localization of cyclooxygenase isoforms and cytosolic phospholipase A2 in anti-Thy-1 glomerulonephritis. *J. Am. Soc. Nephrol.* **9,** 408–416.
91. Komers, R., Lindsley, J.N., Oyama, T.T., and Anderson, S. (2007) Cyclooxygenase-2 inhibition attenuates the progression of nephropathy in uninephrectomized diabetic rats. *Clin. Exp. Pharmacol. Physiol.* **34,** 36–41.
92. Komers, R., Lindsley, J.N., Oyama, T.T., Schutzer, W.E., Reed, J.F., Mader, S.L., and Anderson, S. (2001) Immunohistochemical and functional correlations of renal cyclooxygenase-2 in experimental diabetes. *J. Clin. Invest.* **107,** 889–898.
93. Wang, J.L., Cheng, H.F., Zhang, M.Z., McKanna, J.A., and Harris, R.C. (1998) Selective increase of cyclooxygenase-2 expression in a model of renal ablation. *Am. J. Physiol*. **275,** F613–622.
94. Ishibashi, R., Tanaka, I., Kotani, M., Muro, S., Goto, M., Sugawara, A., Mukoyama, M., Sugimoto, Y., Ichikawa, A., Narumiya, S., and Nakao, K. (1999) Roles of prostaglandin E receptors in mesangial cells under high-glucose conditions. *Kidney Int.* **56,** 589–600.
95. Mene, P. and Dunn, M.J. (1986) Contractile effects of TxA2 and endoperoxide analogues on cultured rat glomerular mesangial cells. *Am. J. Physiol.* **251,** F1029–1035.
96. Bek, M., Nusing, R., Kowark, P., Henger, A., Mundel, P., and Pavenstadt, H. (1999) Characterization of prostanoid receptors in podocytes. *J. Am. Soc. Nephrol.* **10,** 2084–2093.
97. Makino, H., Tanaka, I., Mukoyama, M., Sugawara, A., Mori, K., Muro, S., Suganami, T., Yahata, K., Ishibashi, R., Ohuchida, S., Maruyama, T., Narumiya, S., and Nakao, K. (2002) Prevention of diabetic nephropathy in rats by prostaglandin E receptor EP1-selective antagonist. *J. Am. Soc. Nephrol.* **13,** 1757–1765.
98. Faour, W.H., Gomi, K., and Kennedy, C.R. (2008) PGE(2) induces COX-2 expression in podocytes via the EP(4) receptor through a PKA-independent mechanism. *Cell. Signal.* **20,** 2156–2164.
99. Craven, P.A., Melhem, M.F., and DeRubertis, F.R. (1992) Thromboxane in the pathogenesis of glomerular injury in diabetes. *Kidney Int.* **42,** 937–946.
100. Matsuo, Y., Takagawa, I., Koshida, H., Kawabata, T., Nakamura, M., Ida, T., Zhou, L., and Marumo, F. (1995) Antiproteinuric effect of a thromboxane receptor antagonist, S-1452, on rat diabetic nephropathy and murine lupus nephritis. *Pharmacology* **50,** 1–8.
101. Sebekova, K., Eifert, T., Klassen, A., Heidland, A., and Amann, K. (2007) Renal effects of S18886 (Terutroban), a TP receptor antagonist, in an experimental model of type 2 diabetes. *Diabetes* **56,** 968–974.
102. Xu, S., Jiang, B., Maitland, K.A., Bayat, H., Gu, J., Nadler, J.L., Corda, S., Lavielle, G., Verbeuren, T.J., Zuccollo, A., and Cohen, R.A. (2006) The thromboxane receptor antagonist S18886 attenuates renal oxidant stress and proteinuria in diabetic apolipoprotein E-deficient mice. *Diabetes* **55,** 110–119.
103. Issemann, I. and Green, S. (1990) Activation of a member of the steroid hormone receptor superfamily by peroxisome proliferators. *Nature* **347,** 645–650.
104. Berger, J. and Moller, D.E. (2002) The mechanisms of action of PPARs. *Annu. Rev. Med.* **53,** 409–435.
105. Kumar, S., Boulton, A.J., Beck-Nielsen, H., Berthezene, F., Muggeo, M., Persson, B., Spinas, G.A., Donoghue, S., Lettis, S., and Stewart-Long, P. (1996) Troglitazone, an insulin action enhancer, improves metabolic control in NIDDM patients. Troglitazone Study Group. *Diabetologia* **39,** 701–709.
106. Campbell, I.W. (2005) The clinical significance of PPAR gamma agonism. *Curr. Mol. Med.* **5,** 349–363.
107. Lennon, R., Welsh, G.I., Singh, A., Satchell, S.C., Coward, R.J., Tavare, J.M., Mathieson, P.W., and Saleem, M.A. (2009) Rosiglitazone enhances glucose uptake in glomerular podocytes using the glucose transporter GLUT1. *Diabetologia* **52,** 1944–1952.
108. Benigni, A., Zoja, C., Tomasoni, S., Campana, M., Corna, D., Zanchi, C., Gagliardini, E., Garofano, E., Rottoli, D., Ito, T., and Remuzzi, G. (2006) Transcriptional regulation of nephrin gene by peroxisome proliferator-activated receptor-gamma agonist: molecular mechanism of the antiproteinuric effect of pioglitazone. *J. Am. Soc. Nephrol.* **17,** 1624–1632.
109. Fukami, K., Yamagishi, S., Ueda, S., and Okuda, S. (2008) Role of AGEs in diabetic nephropathy. *Curr. Pharm. Des.* **14,** 946–952.

110. Wendt, T.M., Tanji, N., Guo, J., Kislinger, T.R., Qu, W., Lu, Y., Bucciarelli, L.G., Rong, L.L., Moser, B., Markowitz, G.S., Stein, G., Bierhaus, A., Liliensiek, B., Arnold, B., Nawroth, P.P., Stern, D.M., D'Agati, V.D., and Schmidt, A.M. (2003) RAGE drives the development of glomerulosclerosis and implicates podocyte activation in the pathogenesis of diabetic nephropathy. *Am. J. Pathol.* **162,** 1123–1137.
111. Doublier, S., Salvidio, G., Lupia, E., Ruotsalainen, V., Verzola, D., Deferrari, G., and Camussi, G. (2003) Nephrin expression is reduced in human diabetic nephropathy: evidence for a distinct role for glycated albumin and angiotensin II. *Diabetes* **52,** 1023–1030.
112. Figarola, J.L., Scott, S., Loera, S., Tessler, C., Chu, P., Weiss, L., Hardy, J., and Rahbar, S. (2003) LR-90 a new advanced glycation endproduct inhibitor prevents progression of diabetic nephropathy in streptozotocin-diabetic rats. *Diabetologia* **46,** 1140–1152.
113. Peppa, M., Brem, H., Cai, W., Zhang, J.G., Basgen, J., Li, Z., Vlassara, H., and Uribarri, J. (2006) Prevention and reversal of diabetic nephropathy in db/db mice treated with alagebrium (ALT-711). *Am. J. Nephrol.* **26,** 430–436.
114. Kim, Y.S., Kim, J., Kim, C.S., Sohn, E.J., Lee, Y.M., Jeong, I.H., Kim, H., Jang, D.S., and Kim, J.S. (2009) KIOM-79, an inhibitor of AGEs-protein cross-linking, prevents progression of nephropathy in Zucker diabetic fatty rats. *Evid. Based Complement. Alternat. Med.* [Epub ahead of print]
115. Ruster, C., Bondeva, T., Franke, S., Tanaka, N., Yamamoto, H., and Wolf, G. (2009) Angiotensin II upregulates RAGE expression on podocytes: role of AT2 receptors. *Am. J. Nephrol.* **29,** 538–550.
116. Sharma, K., Ramachandrarao, S., Qiu, G., Usui, H.K., Zhu, Y., Dunn, S.R., Ouedraogo, R., Hough, K., McCue, P., Chan, L., Falkner, B., and Goldstein, B.J. (2008) Adiponectin regulates albuminuria and podocyte function in mice. *J. Clin. Invest.* **118,** 1645–1656.
117. Tsioufis, C., Dimitriadis, K., Chatzis, D., Vasiliadou, C., Tousoulis, D., Papademetriou, V., Toutouzas, P., Stefanadis, C., and Kallikazaros, I. (2005) Relation of microalbuminuria to adiponectin and augmented C-reactive protein levels in men with essential hypertension. *Am. J. Cardiol.* **96,** 946–951.
118. Tao, L., Gao, E., Jiao, X., Yuan, Y., Li, S., Christopher, T.A., Lopez, B.L., Koch, W., Chan, L., Goldstein, B.J., and Ma, X.L. (2007) Adiponectin cardioprotection after myocardial ischemia/reperfusion involves the reduction of oxidative/nitrative stress. *Circulation* **115,** 1408–1416.
119. Ohashi, K., Iwatani, H., Kihara, S., Nakagawa, Y., Komura, N., Fujita, K., Maeda, N., Nishida, M., Katsube, F., Shimomura, I., Ito, T., and Funahashi, T. (2007) Exacerbation of albuminuria and renal fibrosis in subtotal renal ablation model of adiponectin-knockout mice. *Arterioscler. Thromb. Vasc. Biol.* **27,** 1910–1917.
120. Ahima, R.S. (2008) Linking adiponectin to proteinuria. *J. Clin. Invest.* **118,** 1619–1622.
121. Cammisotto, P.G. and Bendayan, M. (2008) Adiponectin stimulates phosphorylation of AMP-activated protein kinase alpha in renal glomeruli. *J. Mol. Histol*. **39,** 579–584.
122. Kato, M., Arce, L., and Natarajan, R. (2009) MicroRNAs and their role in progressive kidney diseases. *Clin. J. Am. Soc. Nephrol.* **4,** 1255–1266.
123. Shi, S., Yu, L., Chiu, C., Sun, Y., Chen, J., Khitrov, G., Merkenschlager, M., Holzman, L.B., Zhang, W., Mundel, P., and Bottinger, E.P. (2008) Podocyte-selective deletion of dicer induces proteinuria and glomerulosclerosis. *J. Am. Soc. Nephrol.* **19,** 2159–2169.
124. Harvey, S.J., Jarad, G., Cunningham, J., Goldberg, S., Schermer, B., Harfe, B.D., McManus, M.T., Benzing, T., and Miner, J.H. (2008) Podocyte-specific deletion of dicer alters cytoskeletal dynamics and causes glomerular disease. *J. Am. Soc. Nephrol.* **19,** 2150–2158.
125. Ho, J., Ng, K.H., Rosen, S., Dostal, A., Gregory, R.I., and Kreidberg, J.A. (2008) Podocyte-specific loss of functional microRNAs leads to rapid glomerular and tubular injury. *J. Am. Soc. Nephrol.* **19,** 2069–2065.
126. Sun, Y., Koo, S., White, N., Peralta, E., Esau, C., Dean, N.M., and Perera, R.J. (2004) Development of a micro-array to detect human and mouse microRNAs and characterization of expression in human organs. *Nucleic Acids Res.* **32,** e188.
127. Tian, Z., Greene, A.S., Pietrusz, J.L., Matus, I.R., and Liang, M. (2008) MicroRNA-target pairs in the rat kidney identified by microRNA microarray, proteomic, and bioinformatic analysis. *Genome Res*. **18,** 404–411.
128. Kato, M., Zhang, J., Wang, M., Lanting, L., Yuan, H., Rossi, J.J., and Natarajan, R. (2007) MicroRNA-192 in diabetic kidney glomeruli and its function in TGF-beta-induced collagen expression via inhibition of E-box repressors. *Proc. Natl. Acad. Sci. U. S. A.* **104,** 3432–3437.
